# Iron Oxide/Phosphatic Materials Composites with Potential Applications in Environmental Protection

**DOI:** 10.3390/ma13215034

**Published:** 2020-11-08

**Authors:** Georgiana Cornelia Ispas, Raluca Manea, Roxana Ioana Brazdis, Anda Maria Baroi, Toma Fistos, Radu Claudiu Fierascu, Monica Florentina Raduly

**Affiliations:** 1 National Institute for Research & Development in Chemistry and Petrochemistry–ICECHIM Bucharest, 060021 Bucharest, Romania; georgiana.ispas@icechim.ro (G.C.I.); raluca.manea@icechim.ro (R.M.); anda.baroi@icechim.ro (A.M.B.); toma.fistos@icechim.ro (T.F.); monica.raduly@icechim.ro (M.F.R.); 2 Department of Bioresources and Polymer Science, Faculty of Applied Chemistry and Materials Science, University Politehnica of Bucharest, 011061 Bucharest, Romania; 3 Department of Science and Engineering of Oxide Materials and Nanomaterials, Faculty of Applied Chemistry and Materials Science, University Politehnica of Bucharest, 011061 Bucharest, Romania

**Keywords:** iron oxide/phosphatic materials, analytical characterization, non-steroidal anti-inflammatory drugs removal, photodegradation of dyes, environmental protection

## Abstract

Currently, hydroxyapatite is probably the most researched material, due to its multiple applications in medical, environmental, or cultural heritage, when the classical structure is modified and calcium is displaced partially or totally with different metals. By changing the classical structure of the hydroxyapatite, new morphologies can be obtained, thus allowing final applications different from those of the initial hydroxyapatite material. However, their properties should be tuned for the desired application. In this context, the present paper describes the synthesis and characterization (through energy-dispersive X-ray fluorescence, X-ray diffraction, FTIR, thermal analysis, and transmission electron microscopy) of iron oxide/manganese-containing phosphatic phase composite materials, developed in order to obtain the enhancement of final environmental applications (photodegradation of dyes, adsorption of organic compounds). The composite material was tested for photocatalytic properties, after embedding in hydrosoluble film-forming materials. Photocatalytic coatings show different activity during the photodecomposition of Methylene Blue, used as a model of a contaminant. The photocatalytic activities of the materials were discussed in relationship with both the phosphatic materials and the magnetic components. Finally, other environmental applications were studied for the developed materials (adsorption of non-steroidal anti-inflammatory drugs—paracetamol and ibuprofen), revealing an enhancement of the adsorption capacity of the phosphatic material upon addition of the magnetic phase.

## 1. Introduction

The whole world is facing drinking water deficiencies due to groundwater contamination and untreated or partially treated wastewater discharges. The demand for drinking water is increasing day by day due to the exponential growth of the population and has become a severe problem that must be solved as a priority. As a result of industrialization and urbanization, the environment and human health can suffer from possible negative effects due to the resulting hazardous compounds, which must be treated before their discharge into the water streams [1,2,3].

As the problem of environmental pollution worsens day by day, attempts have been made to remove pollutants, such as organic substances (phenols [4] and dyes [5]) and inorganic substances, such as heavy metals [6,7], using hydroxyapatite, from wastewater and soil, which seems to be much more promising, in terms of its properties, but also of the low costs involved in its synthesis and application [8,9,10] compared with other compounds such as magnetic hydrogels [8] or chitosan-calcium alginate blended beads [9]. Among the appropriate solutions for removing and reducing the proportion of heavy metals in groundwater are membrane isolation, ion exchange, reverse osmosis, chemical precipitation, and electrolysis, whose choice depends on the type and concentration of both sorptive and sorbent material used and on their cost. These methods proved to be expensive and incompetent, particularly in the removal of trace amounts of heavy metals. The process of adsorption is most widely used, since adsorption is an important technique for removing different contamination from aqueous solutions. Adsorption efficiently eliminates pollutants from runoff with heavy solvent loads and also at dilute concentrations. A very high rate of adsorption and excretion occurs during this process [9]. This process has been triggered by basic action as one of the easiest ways to extract iron ions from ground water [11]. In this context, proposing new materials such as apatitic materials can be a good alternative to expensive methods.

In recent years, several papers have reported the use of apatitic materials, with or without a magnetic component, for environmental protection [7,12]. Hydroxyapatite (HAP), with the chemical formula Ca_10_(PO_4_)_6_(OH)_2_, is a phosphate component of hard tissues, such as teeth and human bones, a very promising candidate for solving the problems related to air, water, and soil pollution, with low-cost processes involved in its synthesis, low water solubility, buffering capacity, high stability during oxidation, and adsorption affinity [3]. Therefore, it is considered as a suitable material for the recovery of organic and inorganic pollutants from wastewater. Ionic substitutions (for example, Ag^+^, Sr^2+^, Mn^2+^, Sn^2+^, Co^2+^, Ni^+^, Cu^2+^) considerably improve hydroxyapatite’s properties [13] and enhance the final applications [6].

Moreover, the magnetic phase can be used for functionalization of apatitic materials (with oxides of metals such as Fe, Co, Cu, and Ni), improving the final properties [14]. Magnetic adsorbents are considered to have promising applications in water treatment due to their easily separation under a magnetic field [8].

One concerning problem that still persists in the industrial area is the use and disposal of chemical substances. For example, dyes are commonly used in the cosmetics, food, pharmaceutical, plastic, and textile industries. The treatment of dye-containing wastewater before discharge is very critical. Due to their industrial importance and application, their removal from the environment has been extensively studied. The literature offers various examples considering the removal methods of dyes from wastewaters; worth mentioning is adsorption, advanced oxidation processes, electrochemical degradation, and nanofiltration [15]. Various metal oxides and waste materials are used as an adsorbent, especially activated carbon which is used in large-scale adsorbents [16]. It has been demonstrated that a complete removal of organic dyes is obtained due to the promising photocatalytic activity of HAP [15], which is a stable biomaterial, but also an eco-material photocatalyst suitable for the degradation of persistent pollutants, using UV irradiation [17,18]. Sahoo et al. tried to remove Eriochrome Black T (EBT) from aqueous solutions using hydroxyapatite with a magnetic component (namely ferrous and ferric chloride) [19]. In another study, Piri et al. used magnetic zeolite/hydroxyapatite (MZeo-HAP) as an adsorbent for various dyes in aqueous solutions. Wang et al. synthesized magnetic hydroxyapatite-immobilized oxidized multi-walled carbon nanotubes (mHAP-oMWCNTs) for the adsorption of Pb(II) and methylene blue (MB) from aqueous solutions, the magnetic phase being obtained by the hydrothermal method [20].

Phenolic compounds and non-steroidal anti-inflammatory drugs (NSAIDs) represent another category of pollutants widely found in the environment, especially in wastewaters [4,21]. Our group previously developed some adsorbents based on apatitic materials, substituted with Sr and Ba, which showed good results in terms of stability and potential applications [4]. Bouiahya and co-workers studied alumina-hydroxyapatite composites’ adsorption properties in comparison with pure hydroxyapatite, concluding that their material has better capacities of adsorption than other sorbents (bentonite, zeolites, or alumina) [10]; Karamipour et al. investigated some expensive adsorbents, such as Fe_3_O_4_-coated nanofibers on cellulose acetate and chitosan for Cr(VI), Ni(II), and phenol removal from aqueous solutions [22]; Ekka et al. prepared hydroxyapatite decorated with zirconia nanoparticles (HAp-ZrO_2_) by the sonochemical method and used it in the photodegradation of phenols and its derivates [23]. For NSAIDs removal, activated carbon, ligninolytic enzymes, graphene-based adsorbents, or molecularly imprinted polymers can be used as adsorbents in the depollution process of ground waters or wastewaters [24].

In this context, the present paper describes the synthesis and characterization (through energy-dispersive X-ray fluorescence—XRF, X-ray diffraction—XRD, Fourier-transform infrared spectroscopy - FTIR, thermal analysis, and transmission electron microscopy—TEM) of a composite material, consisting of a magnetic phase and metal-substituted phosphatic (apatitic) materials, as well as its potential use in environmental applications (photodegradation of dyes, adsorption of phenol and non-steroidal anti-inflammatory drugs—paracetamol and ibuprofen). The material used to develop the final composite was selected based on previous preliminary studies [13], the manganese phosphatic material proving to have a good photodegradation efficiency towards methylene blue. Our group studied several metals for developing apatite-type phosphatic materials, manganese raising fewer environmental concerns compared with other metals, such as strontium or barium, for example [25].

## 2. Materials and Methods

### 2.1. Synthesis Method

The composite adsorbent material used in the studies was prepared in a two-stage method. Firstly, the magnetic phase was prepared by coprecipitation, using a previously reported procedure [26]. Briefly, the synthesis was performed in a three-neck flask, under mechanical stirring, by the reaction between FeCl_2_·4H_2_O and FeCl_3_·6H_2_O, in a reaction medium consisting of NH_4_OH (25%) and distilled water, under inert atmosphere, at room temperature. The black precipitate was separated using a magnet after one hour of reaction, washed repeatedly with distilled water, filtered, and dried in vacuum (sample M). In the second phase, the magnetic iron oxide (1.5 g) was dispersed in the distilled water, in which appropriate quantities of Ca(NO_3_)_2_ and MnCl_2_, in a ratio Ca:Mn = 1:1, were subsequently dissolved and the entire solution was brought to 80 °C. The phosphorus containing solution ((NH_4_)_2_HPO_4_), with the pH adjusted to 10 with NH_4_OH, was slowly added into the heated solution, under stirring, maintaining the pH and temperature values at 10 and 80 °C, respectively [6]. The final composite material was separated after approximatively 3 h of reaction, thoroughly and repeatedly washed with distilled water (until a neutral pH was recorded), rinsed with ethanol, and vacuum dried at 45 °C for 48 h. The final weight of the composite material (MnHAP-M) was about 30 g. The MnHAP material (used for comparison purposes in the experiments) was obtained by following the same protocol, without the addition of the magnetic phase, as previously reported [13]. All the reagents used were analytical grade reagents (purity > 98%), supplied by Merck KGaA, Darmstadt, Germany, with the exception of NH_4_OH, supplied by CHIMREACTIV, Bucharest, Romania. The encoding of the samples used in the experiments is presented in Table 1.

### 2.2. Analytical Characterization

X-ray diffraction (Rigaku Corporation, Tokyo, Japan) analyses were performed with a Rigaku SmartLab diffractometer, having the following operating conditions: 45 kV, 200 mA, Cu Kα radiation (1.54059 Å) parallel beam configuration (2θ/θ scan mode), from 5 to 85 2θ degrees; the resulting diffractograms were interpreted using the Rigaku Data Analysis Software PDXL 2 (ver. 2.7.2.0, Rigaku Corporation, Tokyo, Japan), by comparison with ICDD (International Centre for Diffraction Data) database entries. The quantification of the phases present in the composite material was performed with the RIR (Reference Intensity Ratio) method, using the Rigaku Data Analysis Software PDXL 2.

X-ray fluorescence (PANalytical, B.V., Almelo, The Netherlands) measurements were performed using a MiniPal 2 PW4025 (PANalytical) energy-dispersive XRF spectrometer, equipped with a 9W X-ray tube with Rhodium anode; the detector is Si-PIN with beryllium window; measurements were performed in helium atmosphere (for light elements improved sensitivity) for 300 s, at a voltage of 20 kV and automatic current. The quantification of the metals present in the obtained composite (MnHAP-M, in comparison with the manganese phosphatic material MnHAP) was performed using a Vanta C series handheld XRF (Olympus, Waltham, MA, USA), equipped with 40 kV X-ray tube with rhodium anode, Silicon Drift Detector, in the pre-calibrated GeoChem mode, and acquisition time 60 s for each beam. The equipment uses two different energy beams for the quantification of the elements: beam 2 (10 kV) for light elements (Mg, Al, Si, P, S, K, Ca, Ti, Mn) and beam 1 (40 kV) for the rest of the detectable elements.

FTIR spectra of the materials were recorded on a Jasco FTIR 6300 spectrometer (Jasco Corporation, Tokyo, Japan) equipped with an ATR Specac Golden Gate (KRS5 lens), in the range 400–4000 cm^−1^ (30 scans at 4 cm^−1^ resolution).

The morphologies of the synthesized materials were evaluated using a Tecnai G2 F20 TWIN Cryo-TEM transmission electron microscope (FEI Company, Hillsboro, OR, USA) at a 300 kV acceleration voltage with a resolution of 1 Å.

The thermal behavior of the samples was evaluated using a thermogravimetric analysis (TGA) Q5000IR instrument (TA Instruments, New Castle, DE, USA), heating rate 10 °C/min, from room temperature to 1000 °C, in platinum pans, under synthetic air atmosphere (99.999%), at 50 mL/min.

The obtained results were interpreted and graphically presented using OriginPro 2018 data analysis software (ver. 9.50, OriginLab corporation, Northampton, MA, USA).

### 2.3. Photodegradation of Methylene Blue

The potential towards obtaining photocatalytic coatings using the synthesized materials was determined by a generally accepted method, namely UV–vis spectrometry, using methylene blue (MB) as a contaminant. For this experiment, the composite material (MnHAP-M) and the phosphatic material without the magnetic phase (MnHAP, used as reference to study the influence of the magnetic phase) were mixed separately with a styrene-acrylic film-forming material, prepared as previously described [27], using an automatic pigment muller machine (J. Engelsmann AG, Ludwigshafen am Rhein, Germany) in a ratio 1:19 (by weight), to a total weight of three grams [28]. The resulted materials were deposited on glass slides, having a thickness approximatively 50 ± 1 µm, then dried for 48 h. The glass slides were immersed in 30 mL of 1 g/L solution of MB, followed by drying for 2 h at 120 °C and underwent a light fastness testing procedure using a xenon discharge lamp with controlled irradiation (Atlas Xenotest 150S+, Atlas Material Testing Solutions, Mount Prospect, IL, USA).

The reflectance UV–vis spectra on the coatings deposited on glass slides were recorded at 0, 30, 60, 90, 120, and 150 min exposure time using a Jasco V570 spectrometer (Jasco Corporation, Tokyo, Japan) equipped with a Jasco ILN-472 integrating sphere (150 mm) Spectralon as a reference.

The concentration of methylene blue (MB) was measured from the visible diffuse reflectance spectra, while the degradation efficiency was calculated using the equation:

(1) $$DE=\;\frac{\left(R_{t}-\;R_{0}\right)}{R_{t}}\times 100\;$$

Where *DE* is the degradation efficiency (%), *R*_0_ represents the initial reflectance of the coating, and *R_t_* the reflectance at various intervals of exposure to light.

### 2.4. Adsorption Studies

The developed composite material was also evaluated in terms of adsorption capacities towards phenol and two anti-inflammatory drugs (paracetamol and ibuprofen). Adsorption experiments were performed in 15 mL plastic tubes containing 0.2 g of adsorbent and 10 mL pollutant solution at different concentrations (1, 5, 20, 40, 50, 60, 80, 100 mg/L) using an (GFL 3025) shaker (Gesellschaft für Labortechnik Mbh, Burgwedel, Germany) at 20 °C. Distilled water was used for preparing the pollutant solutions. The mixtures were shaken for 24 h in order to reach equilibrium and consequently, the tubes were centrifuged and samples from the supernatant were taken and subjected to high-performance liquid chromatography (HPLC) analysis. HPLC analysis was performed using an L-3000 system (Rigol Technologies Inc., Beijing, China). Analysis conditions were: Kinetex EVO C18 (150 4.6 mm, particle size of 5 μm) column, injection volume of 10 μL; solvents A (30%—0.1% trifluoroacetic acid in water) and B (70%—0.1% trifluoroacetic acid in acetonitrile) were used in isocratic conditions, 220 nm wavelength and 1 mL/min flow rate. As adsorbents, MnHAP and the composite MnHAP-M were used. To the experimental data obtained, several adsorption isotherms models were applied in order to assess the adsorption capacity of solids and some information regarding solid organic compounds interactions.

Several models are known in the literature to model experimental data of adsorption and the most used are Langmuir, Freundlich, Sips, and Temkin. These models are important since they offer insights data concerning adsorbate–adsorbent interaction and evaluate the extent of adsorption. Parameters for each isotherm occur from the fitting process performed on experimental data using user-built functions of Origin software based on isotherm equations:

(2) $$\begin{array}{ccc} \mathrm{Langmuir}\;\mathrm{model}: & \; & q_{e}=\frac{q_{max}bC_{e}}{1+bC_{e}} \end{array}$$

where *q_e_* is the amount of adsorbate adsorbed by 1 g of adsorbent (mg g^−1^), *q_max_* is the maximum single layer adsorption capacity (mg g^−1^), *C_e_* is the amount of adsorbate left in the solution at equilibrium (mg L^−1^), and *b* is a constant related to free energy or adsorption enthalpy (L·mg^−1^).

(3) $$\begin{array}{ccc} \mathrm{Freundlich}\;\mathrm{model}: & \; & q_{e}=K_{f}C_{e}{}^{1/n} \end{array}$$

where *K_f_* (mg·g^−1^) is a constant associated with adsorption capacity and n is an empirical parameter associated with adsorption intensity and shows the strength of the interaction between the phenol and adsorbents. High *K_f_* values show that the adsorbent and the adsorbate are close to each other, while the 𝑛 value indicates the degree of nonlinearity between solution concentration and adsorption: 𝑛 = 1, then adsorption is linear; 𝑛 < 1, then adsorption is a chemical process; 𝑛 > 1, then adsorption is a physical process. Optimal 𝑛 values for a good adsorption process must fall between 1 and 10.

(4) $$\begin{array}{ccc} \mathrm{Temkin}\;\mathrm{model}: & \; & q_{e}=Bln\left(AC_{e\;}\right) \end{array}$$

where *b* is related to heat of adsorption and a is the equilibrium binding constant related to maximum binding energy.

(5) $$\begin{array}{ccc} \mathrm{Sips}\;\mathrm{model}: & \; & q_{e}=\frac{q_{m}{\left(k_{s}C_{e}\right)}^{m}}{1+{\left(k_{s}C_{e}\right)}^{m}} \end{array}$$

where *k_s_* (L mg^−1^)^1/m^ related to energy of adsorption; m—Sips model exponent; *q_ms_* (mg g^−1^)—maximum adsorption capacity

The leakage of iron and manganese from the adsorbent material was analyzed by thoroughly mixing 1 g of composite in 50 mL of distilled water for 24 h, followed by centrifugation and supernatant analysis by the atomic absorption spectroscopy (AAS) method using an atomic absorption spectrophotometer (Solaar M5, Thermo Electron Corporation, Waltham, MA, USA).

For the establishment of the adsorption mechanism, the composite material was characterized, before and after the process, by Raman analysis, carried out using a Renishaw inVia Confocal Raman (Renishaw, Wotton-under-Edge, Gloucestershire, UK) microscope system. The excitation laser wavelength was 473 nm. The Raman spectra were acquired in the extended spectral region from 100 to 3200 cm^−1^, under ambient conditions.

## 3. Results and Discussion

### 3.1. Analytical Characterization of the Materials

As a first step of analysis, the materials were analyzed using X-ray fluorescence, in order to identify any impurities potentially present.

**Figure 1:**
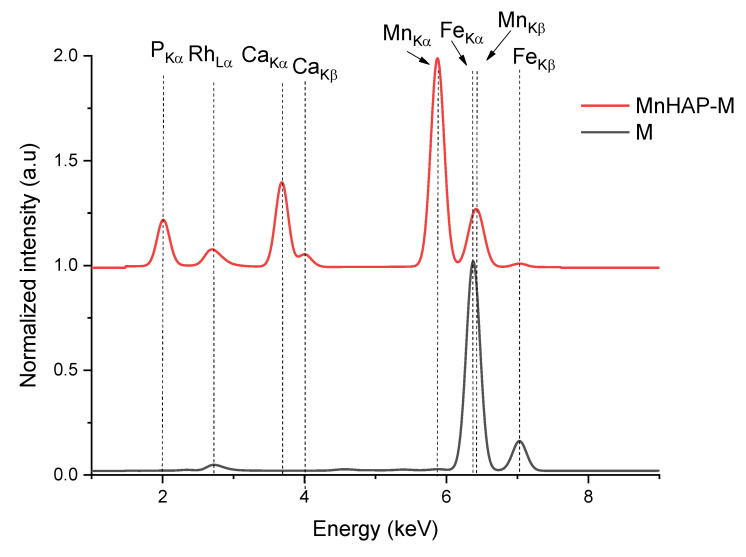
X-ray fluorescence (XRF) spectra of the obtained composite material (MnHAP-M) and of the magnetic iron oxide.

From the XRF spectra (in Figure 1, the region of interest 1–9 keV is presented, as the rest of the spectra did not display any other elements lines), the absence of any impurities in the obtained materials can be noticed. The presence of the magnetic phase in the composite material is suggested by the appearance of peaks specific for iron (K_α_ and K_β_). The influence of iron peaks is visible by the apparition of the K_β_ line (at 7.05 keV) and by the shift recorded by the peak corresponding to the Mn K_β_ line from the MnHAP sample, from 6.49 keV to lower values in the MnHAP-M sample (the peak being centered at 6.42 keV) due to the influence of the Fe K_α_ line (6.4 keV). Elemental composition of the samples (determined by XRF) is presented in Table 2.

**Figure 2:**
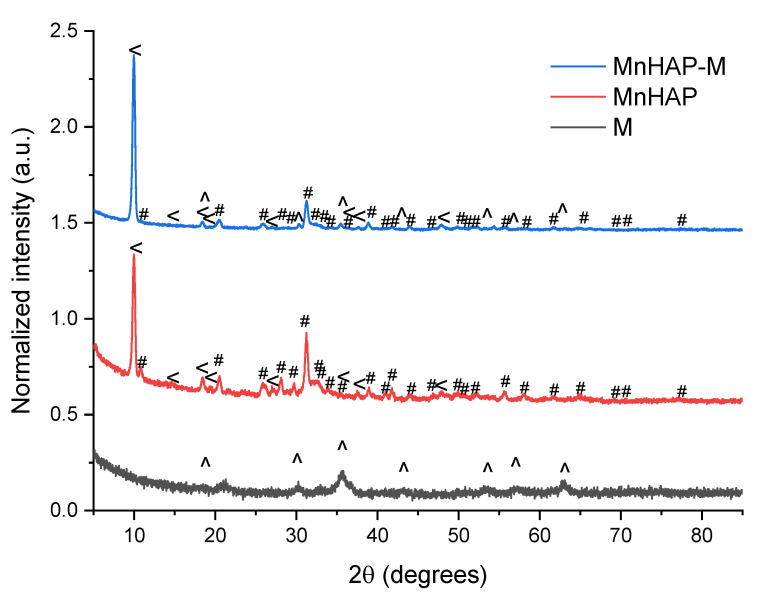
X-ray diffractograms of the analyzed materials. #—peaks corresponding to the manganese apatitic material; <—pararobertsite; ^—iron oxide (magnetite/maghemite).

The phase composition (determined by X-ray diffraction) confirms these findings (Figure 2).

The main peaks that can be associated with the presence of hydroxyapatite and metal-substituted hydroxyapatites (as previously reported by our group) are those appearing at 31.22 degrees, corresponding to the (211) plane, 32.17 degrees—(112), 32.77 degrees (300), and 25.87—(002) [13]. MnHAP also presented a second phosphate phase (presented on Figure 2), consisting of pararobertsite—(Ca_2_(H_2_O)_2_)(Mn_3_O_2_(PO_4_)_3_)(H_2_O), ICDD card no. 01-070-3363, presenting the main peaks at 9.97 degrees—(110), 18.41 degrees—(11-2), 20.53 degrees—(102), and 38.83—(331), respectively. The synthesis of the magnetic phase was confirmed by the apparition of specific magnetite/maghemite XRD peaks at 35.69 degrees—(311) plane, 30.16 degrees—(220), 43.17 degrees—(400), 53.4 degrees (422), 57.15 degrees (511), and 62.70 degrees—(440), respectively. The assignment was made by comparison with the ICDD card no. 01-088-0315. All the detailed peaks are also present in the composite material. Using the RIR method, the quantification of the phases present in the composite material revealed a composition of approx. 37% pararobertsite, 58% apatitic material, and 4% for the magnetic iron oxide, respectively. The results, compared with the MnHAP (approx. 40% for the pararobertsite phase and the rest attributed to the apatitic material), are in good agreement with the XRF determinations. The only difference between the two sets of results is the slightly lower values obtained by XRF for Mn (by comparison with the theoretical calculations based on RIR data), which could be explained by the obtaining of a MnHAP compound with a 1.5 Ca/Mn ratio. However, supplementary experiments are needed for a definitive conclusion.

Another parameter that could be determined from XRD is represented by the magnetic phase particle dimension, important for photodegradation application, as the energy band gap is highly dependent (inversely proportional) on the particle size [29,30]. Using the Scherrer equation [26], the magnetic iron oxide (sample M) crystal size was estimated using the full-width at half-maximum obtained from the (311) plane at approx. 11.1 nm. Estimation of the crystallite size of the manganese-apatitic material in MnHAP was performed using the (211) plane, resulting in dimensions of approx. 18 nm, while the crystallite size of the pararobertsite phase, determined using the (100) peak, was 23.32 nm. In the composite material, the crystallite size of the apatitic material was found to increase to approx. 20.3 nm, of the magnetic phase was found to be 20.13 nm, while the one of the pararobertsite was determined to be 23.15 nm. It can be observed that the apatitic material suffers a small increase in crystallite size, while for the magnetic phase, a strong increase in the crystallite size is observed, most probable due to their transformation in the reaction medium used for the synthesis of the composite material. The obtained values are similar to other studies, presenting the mechanochemical synthesis of magnetite/hydroxyapatite nanocomposites [31].

The FTIR analysis of the synthesized materials presents the specific bands for the apatitic materials: 1089, 1025, 950, and 558 cm^−1^, assigned to the PO_4_^3−^ group. The 1089 and 1025 cm^−1^ peaks are correlated with the symmetric υ_3_ vibration of the PO_4_^3−^ group, the principal peaks for the phosphate vibration modes [6,13]. O–P–O bending vibrations (between 605 and 550 cm^−1^) are also observed in the FTIR spectra [6]. The IR spectra of sample M are dominated by the bands in 630–530 cm^−1^, which can be attributed, as other authors presented, to the Fe–O bonds vibrations in tetrahedral and octahedral sites, resulting from the split of the ν_1_ band from 570 cm^−1^ [32,33]. The band at 443 cm^−1^ is due to octahedral Fe, corresponding to the Fe–O ν_2_ band [32], while the peak at approx. 515 cm^−1^ is considered as a characteristic peak of magnetite, according to the literature data [33]. In all the samples, the O-H stretching vibration (approx. 3420 cm^−1^) and deformed vibration (approx. 1640 cm^−1^) are due to the presence of coordinated OH groups or water molecules [32].

**Figure 3:**
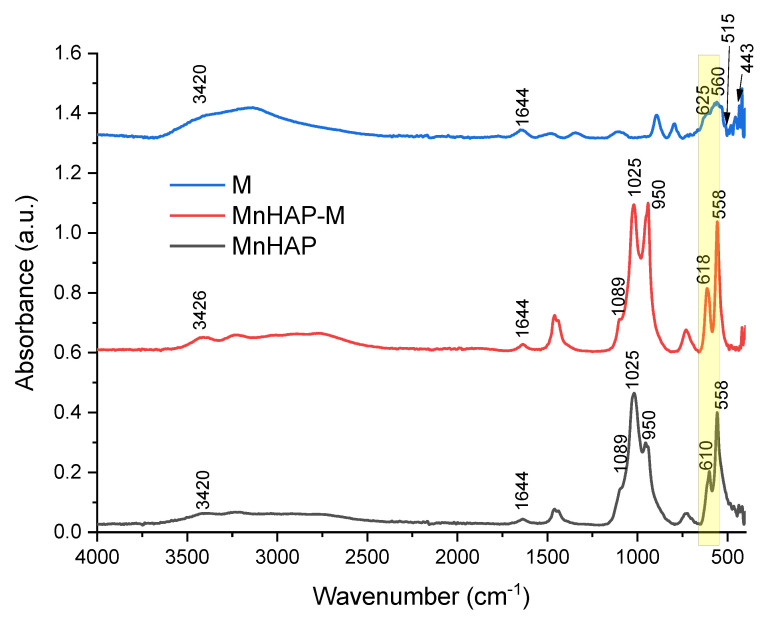
FTIR spectra of the composite material (MnHAP-M), compared with the manganese phosphatic material (MnHAP) and the magnetic iron oxide (M). Peaks further discussed are detailed on the spectra.

The magnetic phase in the MnHAP-M composite is suggested by the change in spectra in the region 630–550 cm^−1^ (highlighted in yellow on Figure 3), which can be attributed to the vibrations of Fe–O bonds in tetrahedral and octahedral sites [32]. Due to the low concentration of magnetic phase in the final product, as well as considering the overlapping with the bands specific to the apatitic material, it was expected that the magnetic phase was hardly identifiable by FTIR. However, the presence of magnetite in a nanocomposite material was determined similarly by analyzing their FTIR spectra [34].

**Figure 4:**
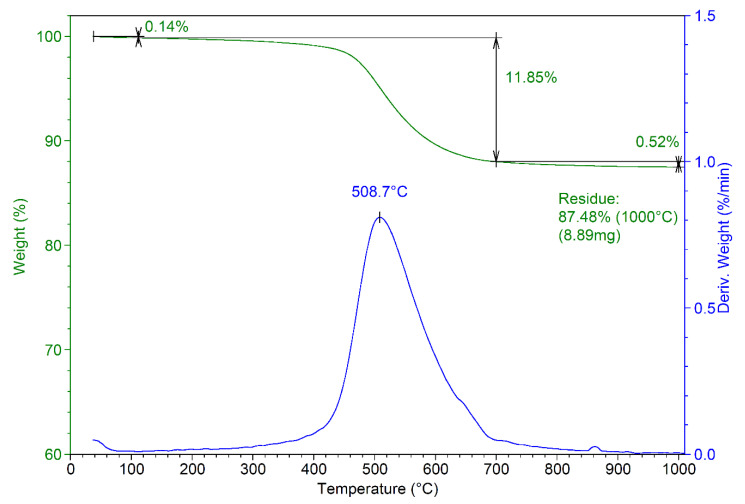
Thermogram of the developed composite material (MnHAP-M).

The thermal analysis of the obtained composite material is presented in Figure 4. The thermogram reveals a residue at 1000 °C of approximately 87.5%, compared with 84.7 for MnHAP (thermogram not presented) [13]. The mass losses recorded for the composite can be divided into three stages: stage 1—from room temperature to about 110 °C (mass loss 0.14%), associated with the desorption of adsorbed water from the surface of the powders [35]; stage 2—from 110 to 700 °C (mass loss 11.85%, T_max_ = 508.7 °C), associated with the loss of lattice water [36]. This stage presents the main weight loss for the composite materials, as in this region, other phenomena also appear, including the dehydroxylation of the P-OH groups (formed by the reactive surface phosphorus) to P–O–P surface groups at temperatures above 400 °C [37]. Finally, the last stage (above 700 °C) records a relatively small mass loss (0.52%). This can be associated with the dehydroxylation of the apatitic materials, which begins at around 850–900 °C and should end at higher temperatures with the formation of the corresponding oxy-form of apatitic materials [37].

**Figure 5:**
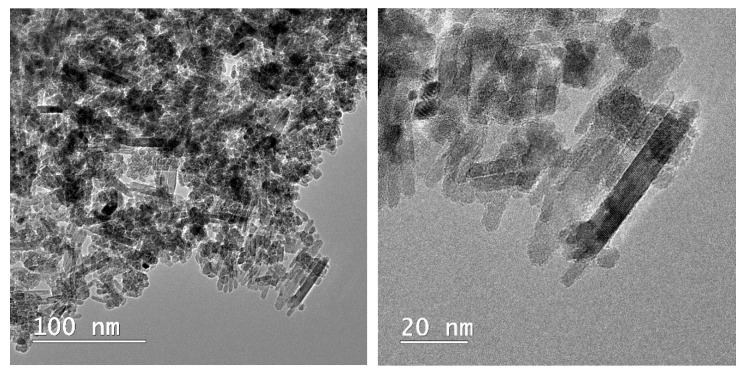
TEM images of the composite material (MnHAP-M), at different magnifications.

Microscopical observations on the composite material are presented in Figure 5. The rod-shaped structure of the apatitic material can be noticed, as presented by our group [6,13] and other authors for metal-substituted hydroxyapatite [38], as well as the presence of the smaller, quasi-spherical magnetic phase. The overall morphology of the composite is similar to the one reported by Kermanian et al. [39] for magnetite nanoparticles dispersed on hydroxyapatite nanorods. Given the identified morphologies on the TEM images, it is most likely that the pararobertsite phase also has a rod-shaped structure, but this aspect remains to be fully elucidated.

### 3.2. Photodegradation Studies

**Figure 6:**
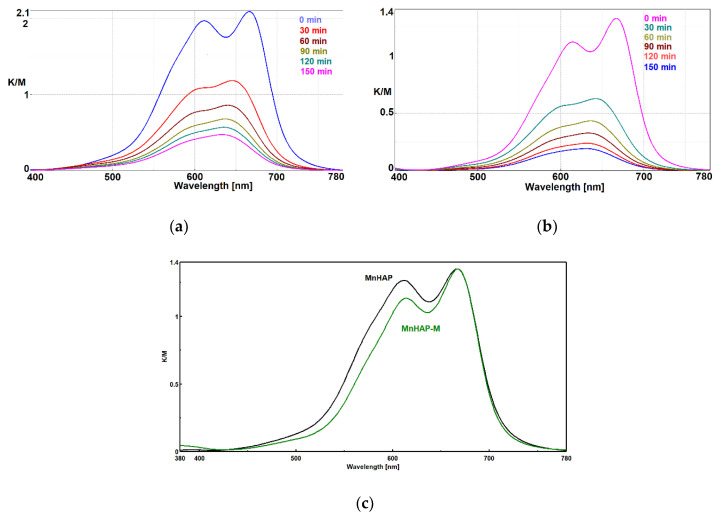
Photodecomposition of MB using coatings containing HAP-Mn (**a**) and HAP-Mn-M (**b**) at 0, 30, 60, 90, 120, and 150 min.; normalized Kubelka–Munk spectra for the two used materials (**c**).

The absorption spectrum of MB presents a distinct band at 664 nm, attributed to the auxochromic group of MB. The decrease in intensity of this peak (as observed in Figure 6) is related to the MB bleaching/degradation of this specific group [40].

In the case of MnHAP-M, the decrease in intensity of the peak at 605 nm shows that MB is distributed mainly at the molecular level onto the surface of the photocatalyst, which is an advantage for the photocatalytic process and indirect evidence of the higher surface area obtained for the catalyst containing a magnetic phase. Probably, the quasi-spherical magnetic particles prevent the aggregation of the rod-shaped HAP nanoparticles, increasing the surface area.

In order to clarify the problem of the existence of MB monomer-dimer species on the surface of photocatalytic composites, the Kubelka–Munk spectra were normalized, as shown in Figure 6c.

It is obvious that in the case of composites with the magnetic phase, there is a decrease in the peak corresponding to the dimeric MB species, which shows us that under identical conditions of MB concentration in the impregnation solution, adsorption of the dye at the molecular level on a larger surface is obtained due to the existence of the spherical magnetic nanoparticles. Spherical nanoparticles, as seen from TEM, provide an increase in the surface area of MnHAP nanoparticles with which they come into contact, making it impossible to aggregate them on edges and faces, a process that decreases the surface area of photocatalytic composites in MnHAP. The spherical morphology of the magnetic phase is optimal in terms of contact (at one point) with MnHAP nanoparticles, for it exposes new faces and edges available for monomolecular adsorption of MB.

**Figure 7:**
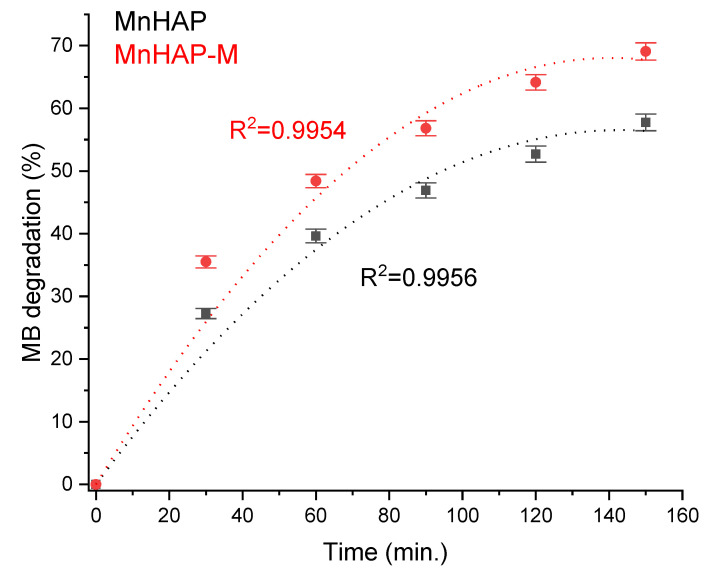
MB photodegradation efficiency under xenon light exposure.

Photodegradation efficiency was enhanced by the addition of the magnetic phase in the composite material (Figure 7). The obtained results are promising towards further practical applications, considering the observations present in the literature data (especially considering the low concentration of the composite material in the tested coating), such as those obtained for nanostructured hydroxyapatite spheres (tested as powder material, 75% degradation) [41], hydroxyapatite from natural sources (powder tested at 2 g/L concentration, 62% degradation observed in 24 h) [42], hydroxyapatite nested bundles (0.5 g/L powder, observed degradation 77% after 210 min) [43], metal-doped titania/HAP composites (1 g/L powder, MB degradation 67–91%) [44], titania/HAP/reduced graphene oxide composites (coating containing 10% active compound, 78% degradation of methylene blue after 5.5 h of exposure) [45], nanocrystals of titanium-substituted hydroxyapatite (5 g/L powder, 50% MB degradation after 1 h) [46], or hydroxyapatite/α-silver vanadate (1 g/L powder, 85% MB degradation observed in 60 min) [47].

The increase in photodegradation efficiency for the composite material is most likely correlated with a mechanism similar to the “absorb and shuttle” mechanism. Thus, using an absorbent substrate (in our case, MnHAP, also possessing photocatalytic properties) leads to the concentration of the pollutant around the photocatalyst, resulting in an enhancement of the photodegradation efficiency. The sample MnHAP also presents relatively good photodegradation efficiency, following a similar mechanism to the one described by Shariffuddin et al. [42].

The Mn(II) ion has a smaller radius than Ca (II) and the introduction of this metal ion in the hydroxyapatite matrix leads to a structural disorder of the initial cell of HAP. As a consequence, the appearance of intermediate energetic states between the valence and conduction bands allow the usage of visible light to promote electrons from VB (valence band) to the intermediate energetic states, in connection with the formation of vacancies which react with water that is present on the surface of HAP and generate strongly oxidizing hydroxyl radicals.

Moreover, oxidation of Mn(II) to Mn(III) and Mn(IV), explained by the oxygen adsorption and oxidation reaction, generate superoxide anion radicals. As a result, the enhanced MB removal activity of Mn-HAP nanocomposites during visible light irradiation is probably due to the interplay of redox-active Mn ions and the intrinsic charge compensating defects.

The mechanism of MB photocatalytic degradation was extensively studied [48,49] and some of the intermediates formed during the reaction were identified. In order to summarize, photodegradation takes place due to the strongly oxidizing hydroxyl radicals either at the sulfur or nitrogen atoms, as follows:

Via the sulfur atom:
  (a) The hydroxyl radicals attack at the sulfur atom and generate a sulfoxide group and determine the opening of the central ring (containing S and N heteroatoms);
  (b) A second attack due to the hydroxyl radicals produces a sulfone group and determines the definitive dissociation of the two benzene rings;
  (c) A third attack on sulfones generates the sulfonic acids;
  (d) Finally, sulfonic groups are transformed into sulfate ions.
Via the nitrogen atoms:
  (a) The amino groups obtained by the cleavage of the central imino group, can be substituted by a hydroxyl radical, forming the corresponding phenol and generating ammonia and ammonium ions, which are finally oxidized to nitrates;
  (b) Radical hydroxyl undergoes a progressive oxidation of methyl groups belonging to the dimethyl-phenyl-amino groups, forming alcohol and subsequently, acids, which are decarboxylated to carbon dioxide.

The results obtained suggest the possibility to develop cheaper, efficient photocatalysts, using readily available materials and a simple synthesis procedure.

### 3.3. Batch Adsorption Isotherm

**Figure 8:**
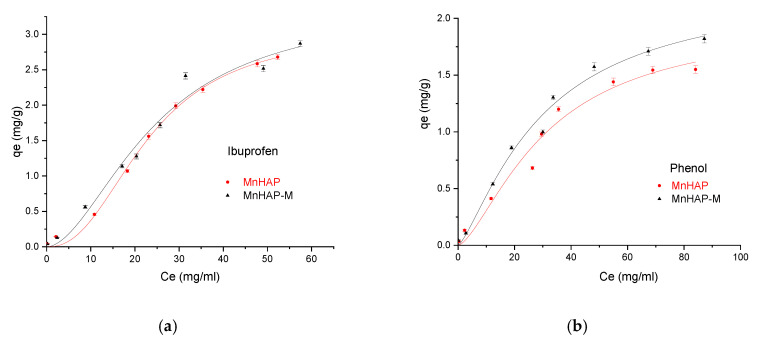
Influence of the adsorbent type in the adsorption process using ibuprofen as pollutant (**a**), and phenol (**b**), respectively.

**Figure 9:**
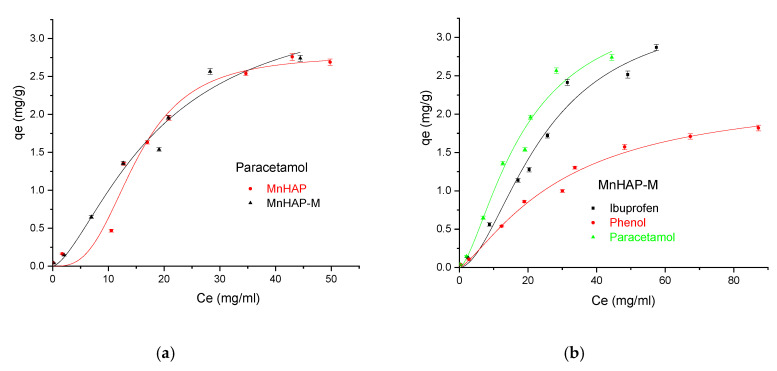
(**a**) Influence of the adsorbent type in the adsorption process using paracetamol as pollutant; (**b**) Influence of the pollutant type in the adsorption process using the MnHAP-M composite material.

Finally, the developed materials were tested for pollutant adsorption in batch experiments. The experimental data obtained were fitted according to the Sips model presented in Figure 8 and Figure 9 and all parameters obtained from the Langmuir, Freundlich, Sips, and Temkin models are summarized in Table 3.

Adsorption isotherms cope with equilibrium data and are important envisage the opportunity of obtaining the optimal parameters for the process. The quantity of pollutants adsorbed on the solid surface at equilibrium was calculated by the equation:

(6) $$\;q_{e}=\frac{\left(C_{0}-C_{e}\right)\cdot V}{m}$$

Among the four models used (as presented in Section 2.4. Adsorption studies), the Sips model (describing the adsorption process on heterogeneous surfaces) better fits experimental data having higher R^2^ regardless of the pollutant and solid used. Hence, on the basis of these error functions, the following trend was observed for different models:

(7) Sips>Langmuir≫Freundlich>Temkin

In the Sips isotherm, surface heterogeneity is given by parameter *m* and is useful to characterize the adsorbent surface (in our case, suggesting a heterogenous surface). Surprisingly, the parameter is closer to the unit for the composite material, suggesting that the addition of the magnetic phase in the MnHAP synthesis led to the obtaining of a more homogenous surface, compared with the simple MnHAP (which presents the secondary phase, affecting the surface homogeneity).

The MnHap-M composite shows adsorption capacity of about 6.80 mg/g for ibuprofen, 5.64 mg/g for paracetamol, and 2.90 mg/g for phenol, respectively (higher compared with the sample MnHAP for phenol and paracetamol). The adsorption capacities of the tested materials towards ibuprofen are lower than classical adsorbents, such as activated carbon (which registered adsorption capacities of approximately 140 mg/g) [50], but comparable with the adsorption capacities of activated carbon from natural sources, whose adsorption capacities recorded by the literature studies were around 16 [51] and 9 mg/g [52], respectively, but superior to some clay minerals (such as montmorillonite or kaolinite—approximately 6 and 3 mg/g, respectively) [53]. The same observations can be made regarding the adsorption of paracetamol, presenting superior results compared with activated carbon (0.15 mg/g) [54], but lower than magnetite/activated carbon composites (142 mg/g) [55]. The developed materials possess higher adsorption capacities compared with nano-hydroxyapatite tested against some other types of pharmaceutical pollutants (such as norfloxacin and ciprofloxacin) [56]. Regarding the phenol adsorption, the values obtained are comparable with those obtained by using HAP nanopowders (approximately 10 mg/g, but working at high temperatures—60 °C) [57] or magnetic hydroxyapatite (18 mg/g) [58].

**Figure 10:**
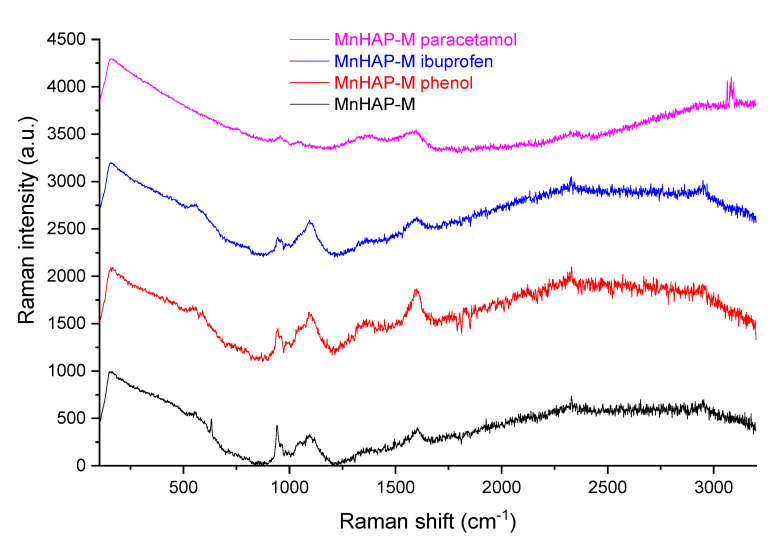
Raman spectra of the composite material before and after the adsorption process.

In order to propose a mechanism for pollutant adsorption on the composite, Raman spectra were recorded before and after the adsorption process (Figure 10).

As can be seen from Figure 10, the Raman spectra of MnHap-M is considerably changed after adsorption of phenol, ibuprofen, and paracetamol. From the changing of the characteristic apatitic bands (the vibration modes of the PO_4_ unit at 560 cm^−1^—antisymmetric bending mode; 960 cm^−1^—symmetric stretching mode; 1040 cm^−1^—antisymmetric stretching mode; 628 cm^−1^—degenerate bending mode ν4 of the PO_4_ group—O−P−O bond, respectively [59,60]), it can be suggested that interaction of pollutants with phosphate groups diminishes the adsorption band, thus supporting a mechanism involving the interaction of pollutants with these specific groups (especially visible in the case of the two non-steroidal anti-inflammatory drugs—paracetamol and ibuprofen—for which adsorption capacities were significantly higher, compared with the one obtained for phenol).

The leaching of iron and manganese was evaluated using AAS (results presented in Table 4).

As visible from Table 4, the leaching was undetectable for iron, while manganese presented a minor leaching (under 1 mg/L, representing under 1% leaching from the total manganese content of the sample).

## 4. Conclusions

In this study, a composite material (Mn-substituted apatitic material with a magnetic phase) was obtained and characterized. The model dye degradation by the photocatalytic coatings (containing the composite material) recommends it as a potential candidate for decorative coatings, which can lead to a decrease in environmental pollution, as well as fungal and algae growth, as the composite material presented a MB degradation efficiency of approximately 70% after 150 min (higher compared with MB degradation efficiency of the phosphatic material, without the magnetic phase— approximately 58% after 150 min). The adsorption capacity of the composite material was tested toward harmful environmental pollutants like ibuprofen, paracetamol, and phenol, by comparison with the Mn-substituted apatitic material. The adsorption ability depends strongly on the nature of the pollutant and the maximum capacity for the composite adsorbent was found to be 6.80 mg/g for ibuprofen, 5.63 mg/g for paracetamol, and 2.90 mg/g for phenol, respectively, suggesting potential applications as an inexpensive adsorbent for the removal of such pollutants. Additionally, the composite presented a minor leaching of the component phases (as proven by the analysis of the supernatant. Mechanisms for both the photocatalytic degradation and the pollutant adsorption using the developed composite were proposed. As a potential perspective, magnetic phases can be used in industrial applications due to their potential to enhance the photocatalytic and adsorbent properties of the original material, as well as to their easy removal. Regarding the application of the proposed material as a depollution material, the challenges are in the synthesis stages, when it is essential to develop nanomaterials with controlled morphologies, tunable shape and size, and easy modification or functionalization. Thus, environmentally friendly and low-cost purification options can be used with excellent potential to remove different classes of contaminants from waste waters.

## Figures and Tables

**Table 1:** Sample encoding.

Encoding	Description
M	Magnetic iron oxide
MnHAP	Mn-containing phosphatic material
MnHAP-M	Composite material: Mn-containing phosphatic material with magnetic iron oxide

**Table 2:** Elemental analysis of the MnHAP and MnHAP-M samples. L.E.—light elements (not detectable by XRF).

Element	MnHAP (%)	MnHAP-M (%)
Mn	23.28 ± 0.16	21.68 ± 0.16
Ca	16.91 ± 0.11	14.82 ± 0.11
P	14.54 ± 0.11	14.71 ± 0.11
Fe	0.256 ± 0.02	3.292 ± 0.04
L.E.	45.01 ± 0.36	45.49 ± 0.48

**Table 3:** Parameters obtained from the Langmuir, Freundlich, Sips, and Temkin models.

Pollutant	Ibuprofen		Phenol		Paracetamol	
Adsorbent	MnHAP	MnHAP-M	MnHAP	MnHAP-M	MnHAP	MnHAP-M
**Langmuir**						
*q_max_* (mg g^−1^)	9.5832	6.8066	2.7821	2.9012	5.5192	5.6394
*b* (L mg^−1^)	0.0079	0.0129	0.0173	0.0212	0.0222	0.0237
R^2^	0.9749	0.9652	0.9685	0.9839	0.9414	0.9667
χ^2^	0.0265	0.0382	0.0112	0.0071	0.0681	0.0355
**Freundlich**						
*K_F_* (mg^1−1/n^ L^1/n^ g^−1^)	0.1027	0.1468	0.1158	0.1526	0.2195	0.2230
n	1.1913	1.3449	1.6476	1.7476	1.4978	1.4602
R^2^	0.9676	0.9526	0.9511	0.9604	0.9224	0.9456
χ^2^	0.0344	0.0521	0.0173	0.0175	0.0902	0.0581
**Temkin**						
α (L mg^−1^)	2.9013	1.5116	1.3014	2.0275	2.7231	2.0886
β (kJ mol^−1^)	0.4101	0.4946	0.2912	0.2943	0.4643	0.4913
R^2^	0.6211	0.6842	0.7842	0.7474	0.6751	0.7154
χ^2^	0.4026	0.3469	0.0768	0.1118	0.3778	0.3039
**Sips**						
*q_m_* (mg g^−1^)	3.0571	3.4247	1.9754	2.2858	2.7855	3.5723
*K_S_* (L mg^−1^)^1/m^	0.0434	0.0410	0.0323	0.0337	0.0667	0.0535
M	2.3672	1.8144	1.5007	1.3263	3.0104	1.5241
R^2^	0.9957	0.9736	0.9691	0.9858	0.9733	0.9719
χ^2^	0.0043	0.0289	0.0110	0.0063	0.0311	0.0299

**Table 4:** Iron and manganese leaching (as determined by AAS). Results are presented in mg/L. Detection limit (D.L.)—0.1 mg/L for both elements.

Element	Blank Sample	Supernatant from MnHAP-M
Mn	≤ D.L.	0.604 ± 0.01
Fe	≤ D.L.	≤ D.L.
